# *Lactocaseibacillus*-deglycosylated isoflavones prevent Aβ 40-induced Alzheimer’s disease in a rat model

**DOI:** 10.1186/s13568-024-01735-y

**Published:** 2024-08-06

**Authors:** Chin-Feng Liu, Zong-Yang Young, Tsung-Wei Shih, Tzu-Ming Pan, Chun-Lin Lee

**Affiliations:** 1 Continuing Education Program of Food Biotechnology Applications, National Taitung University, Taitung, Taiwan, ROC; 2 Department of Life Science, National Taitung University, 369, Sec. 2, University Rd., Taitung, 95092 Taiwan, ROC; 3 SunWay Biotech Co. LTD., Taipei, Taiwan, ROC; 4 Department of Biochemical Science and Technology, National Taiwan University, Taipei, Taiwan, ROC

**Keywords:** Alzheimer’s disease, *Lactocaseibacillus* fermented soybean milk, Deglycosylated isofavones, Genistein, Daidzein

## Abstract

Alzheimer’s disease (AD) is the most common neurodegenerative disease, with symptoms appearing in the cerebral cortex and hippocampus. amyloid β peptide (Aβ) has been shown to deposit in the brain, causing oxidative stress and inflammation, leading to impaired memory and learning. *Lactocaseibacillus* fermentation can produce deglycosylated isoflavones with high physiological activity, which can scavenge free radicals, enhance total antioxidant capacity and inhibit oxidative inflammatory responses. Therefore, in this study, *Lactocaseibacillus paracasei* subsp. *paracasei* NTU101 (NTU101) fermented soybean milk and its extracts were used as test substances, and AD model rats were established by infusion of Aβ40 in the brain for 28 days, and the preventive and ameliorating effects of NTU 101 fermented soymilk were discussed. Effects of soymilk and unfermented soymilk on AD, and explore its effects on AD. Main functional ingredients. The results showed that deglycosylated isoflavones in NTU101 fermented soybean milk improved AD symptoms. Mechanisms of actions include the inhibition of oxidative inflammation; reduction in the expression of risk factors for tau protein and apo E protein production, the deposition of Aβ40 around the hippocampus, and the expression of TLR-2 and RAGE proteins in astrocytes and microglia; and improvement in the memory and learning ability.

## Key points

1. *Lactocaseibacillus*-fermented soybean milk improved memory and learning abilityof AD rat
2. Deglycosylated isoflavones (genistein and daidzein) were the functional compounds
3. Deglycosylated isoflavones repress Aβ40 deposition by inhibiting TLR 2 and RAGE

## Introduction

Alzheimer’s disease (AD) has gradually become a global health concern (Gao and Li 2023). AD can lead to memory and learning impairment, which, in turn, affect daily activities and communication with others and eventually long-term memory (Jansen et al. 2015). Amyloid beta peptide (Aβ) is involved in the pathogenesis of AD and contributes to the deposition of neurofibrillary tangles (NBTs) and senile plaques (Rahman and Lendel 2021). In addition, Aβ induces oxidative and inflammatory responses, with astrocytes and microglia releasing superoxides to attack nerve cells and cause their death (González-Reyes et al. 2017).

Free radical production can lead to mitochondrial dysfunction and eventually cell death(Sinha et al. 2013). Tumor necrosis factor (TNF)-α, activated B cell nuclear factor kappa light chain enhancer (NF-κB), and interleukin-1β (IL-1β) are substances produced during inflammation. TNF-α activates NF-κB to translate cyclooxygenase-2 (COX-2) and inducible nitric oxide synthase (iNOS) (Lin et al. 2013). COX-2 can dilate blood vessels to increase blood flow, and activated macrophages infect infla med tissues (Medeiros et al. 2010). iNOS induces the production of a large amount of nitric oxide (NO), which can easily combine with oxygen to generate a large number of free radicals that attack cells and tissues, causing damage (Song et al. 2014). IL-1β is a master regulator of inflammation and immune responses, triggering inflammatory responses by inducing COX-2 production (Kitazawa et al. 2008).

During soymilk fermentation, lactic acid bacteria metabolize glycoside isoflavones into aglycone isoflavones, which are highly physiologically active substances that are easily digested and absorbed by the human body. Soy isoflavones have multiple functions. Genistein can bind to estrogen and antagonistic receptors, improving hormone-related symptoms (Sharifi-Rad et al. 2021). Moreover, genistein can regulate axonal growth by stimulating extracellular signal regulated kinase 1/2 and phosphoinositide 3 kinase signaling; protect dopamine cells in mice with 1-methyl-4-phenyl-1,2,3,6-tetrahydropyridine-induced neurodegeneration (Zhao et al. 2015); and improve learning and memory by enhancing synaptic plasticity and increasing cholinergic nervous system activity (Wei et al. 2015).

*Lactocaseibacillus paracasei subsp. paracasei* NTU 101 (NTU 101) has been reported to reduce cholesterol in the liver and blood (Chiu et al. 2006), increase the superoxide dismutase (SOD) level, and reduce the lipid peroxidation index, preventing lipid-induced oxidation injury and atherosclerosis (Tsai et al. 2009). NTU 101 can improve the antigen-presenting ability of dendritic cells, enhance the killing effect of natural killer cells (Tsai et al. 2008), and regulate interleukin 10 (IL-10) and interleukin 12 (IL-12) to strengthen immunity (Chiang and Pan 2012). In this study, we used the Morris water maze test to determine whether feeding with the test substance can improve memory impairment and learning ability in rats with Aβ40-induced AD. Histopathological sections were used to analyze the distribution of proteins in tissues, the expression of oxidative and proinflammatory factors, and the effectiveness of soybean milk fermented with lactic acid bacteria in improving AD. We compared the effects of fermented and unfermented soybean milk and determined the effectiveness of genistin, daidzin, and fermented genistein and daidzin in improving AD. Moreover, we explored differences between the effectiveness of glycosylated isoflavones (genistin and daidzin) and deglycosylated isoflavones (genistein and daidzein) in improving AD.

## Materials and methods

### Chemicals

Beta-amyloid peptide (1–40) was purchased from Tocris Bioscience Co. (Ellisville, MO, USA). 1-step transfer buffer (84731) was purchased from Thermo Fisher Scientific Inc. (Rockford, IL, USA). Ethanol (95%) was purchased from Taiwan Tobacco and Liquor Co. (Taipei, Taiwan). 1,1,3,3-tetramethoxypropane (TMP), 3,3′-Diaminobenzidine, Ethylenediaminetetraacetic acid (EDTA), Ethylene glycol-bis (2-aminoethylether)-*N*,*N*,*N*,*N*-tetraacetic acid (EGTA), Sodium dodecyl sulfate (SDS), Sodium orthovanadate (Na_3_VO_4_), Sodium fluoride (NaF), Thiobarbituric acid (TBA), were purchased from Sigma Chemical Co. (St. Louis, MO, USA). Acrylamide, Ammonium persulfate (APS), Glycine, Hydrochloric acid (HCl), Methanol, Polymer of HRP (horseradish peroxidase), Sodium hydroxide (NaOH), Sulfuric acid (H_2_SO_4_), Trichloroacetic acid (TCA), Tween 20 and Xylene were purchased from Merck KGaA (Millipore) Co. (Darmstadat, Germany). Polyclonal Aβ40 antibody (2454) and Monoclonal Tau protein antibody (4019) were purchased from Cell Signaling Technology Co. (Massachusetts, USA). Polyclonal IL-1β antibody (sc-7884), Polyclonal COX-2 antibody (sc-1747), Monoclonal apo E antibody (sc-6384), Monoclonal NF-κB p65 antibody (sc-8008), Polyclonal IL-6 antibody (sc-1265) were purchased from Santa Cruz Biotechnology, Inc. (Dallas, TX, USA). Polyclonal TNF-α antibody (OABB00178) was purchased from Aviva Systeme Biology Co. (San Diego, Ca, USA). Polyclonal iNOS antibody (PA3-030 A), monoclonal β-actin antibody, goat anti-rabbit IgG, (H + L) antibody Peroxidase Conjugates, goat anti-mouse IgG, (H + L) antibody Peroxidase Conjugates (31430) and BCA protein assay reagent (23225) were purchased from Thermo Fisher Scientific Inc. (Rockford, IL, USA).

### Sample preparation

*Lactocaseibacillus paracasei* subsp. *paracasei* NTU101 is provided by SunWay Biotech Co. LTD. (Taipei, Taiwan, ROC). Soybean milk was prepared by soaking soybeans in distilled water in a ratio of 1:10 (w/v) at room temperature for 8 h and following homogenization with blender, and filtered with gauze to remove the okara. Heat the filtered soy milk to 95 °C for 5 min. Once cooled, it can be stored in the refrigerator at 4 °C for a week. The concentration of soymilk was adjusted to 15Brix before use, and then soaked in a 90 °C water bath for 1 h. After cooling, inoculate with 1% (v/v) activated NTU 101 (cultured from a thawed cryovial from the − 20 °C freezer for 18–24 h) and culture at 37 °C for 48 h. Lyophilized powder was prepared by further centrifugation. Lyophilized powder of NTU 101 (25 g) was extracted with 100 mL of 95% ethanol and placed in a 37 °C water bath for 30 min to prepare the ethanol extract. The alcohol layer was removed and filtered through Whatman No. 42 filter paper. Concentrate with vacuum concentrator. The concentrated extract was then washed with DMSO. It contains 6.26 µg/mg genistein and 6.78 µg/mg daidzein.

### Animals grouping and experiment schedule

Male Sprague Dawley (SD) rats at 6–8 weeks of age were purchased from the BioLasco Co. (Taipei, Taiwan). The animals were housed individually and allowed free access to a standard laboratory chow (Ralston Purina, St Louis, MO, USA) and water. In the experiment, the 48 rats were randomly divided to 8 groups and fed standard chow (control group, NOR; 4.5% fat, 3.34 kcal/g). The animals used in this study were male SD rats, purchased from Lesco Biotechnology Co., Ltd. The experimental group was divided into 6 groups at 8 weeks of age, with 8 animals in each group, for a total of 48 animals. The animals were housed in an environment with a relative humidity of 60%, room temperature of 26 ± 1 °C, a 12-h light cycle from 8:00 to 20:00, and an unrestricted food and water supply. Rats were pre-bred to a body weight of 300 g for experimental grouping and brain infusion pump implantation. They were divided into sham operation group, Vehicle group and Vh group. In the experiment, animals were divided into eight groups of 8 animals each. The sham operation group (Vh group) was injected with blank reagent and had a normal diet. In the Aβ group, β-amyloid peptide containing Aβ40 was injected into the brain for 28 consecutive days and fed with a normal diet. The SmE and L-SmE groups were fed the ethanolic extract of unfermented soybean milk and the ethanolic extract of NTU 101 fermented soybean milk in drinking water, respectively. Genistin and daidzin group (Gs + Dz group) and genistein and daidzein (group Gse + Dze) were added the pure substances to drinking water, respcetively. The dietary dose conversion was calculated according to the formula provided by the US FDA (http://www.fda.gov/cder/cancer/animalframe.htm). Based on a 60 kg adult, 6.25× the recommended daily intake per kg body weight is the test dose per kg in rats. The daily doses for the L-SmE, SME, Gs + Dz, and Gse + Dze groups were 116.4, 116.4, 1.518 (0.729 + 0.789), and 1.518 (0.729 + 0.789) mg/rat/day, respectively.

### Surgery for i.c.v. Aβ40 infusion

Rats were anesthetized with sodium pentobarbital (50 mg/kg BW i.p.). The left skull was exposed and drilled (relative to the bregma; 0.8 mm posterior, 1.4 mm lateral) ac-cording to the atlas of Paxinos and Watson (1982) using a stereotaxic frame (Narishige, To-kyo, Japan). Aβ40 was prepared in the vehicle solvent of 35% (v/v) acetonitrile plus 0.1% (v/v) trifluoroacetic acid (pH 2.0). The osmotic mini-pump (2004, Durect Co., Cupertino, CA, USA) used to result in an animal model of AD with impaired memory was filled with AD solution (24.299 µg Aβ40 and 0.9 mg STZ in 180 µL) or the vehicle solution. The outlet of infusion cannula was inserted 4.0 mm into the left ventricle and attached to the skull with dental cement, and then the mini-pump was quickly implanted into the backs of the rats. AD solution of 180 µL contained in the osmotic pump was continuously infused into left ventricle by 0.28 µL/h for 28 days (Lin et al. 2022).

### Memory and learning ability test

The Morris water maze task was used to evaluate the memory and learning ability from the 22nd day to the 27th day (Lee et al. 2007). A black circular tank (diameter: 140 cm, height: 45 cm) was used as the apparatus of water maze in which a movable escape platform (diameter: 10 cm, height: 25 cm) was located inside the tank. The tank was filled to a height of 27.5 cm with water of temperature approximately 23 °C; thus, the surface of the platform was 2.5 cm below the surface of the water. The circular tank was divided into four quad-rants (I, II, III, and IV), and a position with equal distance from center and edge in the middle of each quadrant was marked for the location of platform. The water tank was located in a test room with many cues external to maze. The room had adjustable indirect light, and camera was set at ceiling above the center of water tank. The position of the cues remained unchanged throughout the water-maze task.

According to the procedure of our previous study (Lee et al. 2007), reference memory test was carried out from the 22nd day to the 24th day and included continuous 4 trials per day. Probe test was immediately carried out after the 12th training trial of reference memory task on the 24th day. Working memory test was performed from the 25th day to the 27th day and consisted of five trails per day.

### Preparation of brains

After completing the behavioral studies, the rats were anesthetized with sodium pentobarbital (65 mg/kg BW, i.p.), and the blood was collected; the cerebral cortex and hippocampus were separated from the whole brain on ice, blotted gently with filter paper to remove blood and extraneous tissue fragments, then flash-frozen with liquid nitrogen and stored at − 80 °C until use. Hippocampus and cortex tissues (100 mg) were crushed with an amalgam mixer (UT-1600, Sharp, Osaka, Japan) and suspended in 1.0 mL of ice-cold Tris saline (50 mM Tris-HCl, pH, 7.6, 0.15 M NaCl) buffer containing 1% (v/v) Tri-ton X-100 and protease inhibitor cocktail, and then sonicated for 30 s. The homogenate was centrifuged at 100,000×*g* for 30 min and the supernatant was used for magnesium analysis using the commercial kit (Fortress Diagnostics Ltd., Antrim, UK). Regarding the protein extraction for immunoblotting, the tissue (100 mg) was homogenated in 1.0 mL of lysis buffer (1% Triton X-100, 20 mM Tris, pH 7.5, 100 mM NaCl, 40 mM NaF, 0.2% SDS, 0.5% deoxycholate, 1 mM EDTA, 1 mM EGTA, and 1 mM Na_3_VO_4_) and brief sonication (10 s). The homogenate was centrifuged at 100,000×*g* for 30 min and the supernatant was used for immunoblotting assay.

### Analysis of lipid peroxidation

The lipid peroxidation analysis method is based on the method of Tarladgis et al. (Tarladgis et al. 1964). Briefly, 1,1,3,3-tetramethoxypropane (TMP) was usd as a standard, TMP was diluted to 1 mM with ultrapure water and then diluted with 1 N H_2_SO_4_ solution to 0, 50, 100, 200, 400, 800, 1000 µM, respectively. Subsequently, 25 µL dilution buffer, 150 µL 5% trichloroacetic acid and 50 µL of 60 mM TBA were mixed well into test tubes. The test tubes are closed and heated in a drybath at 100 °C for 35 min. After cooling, the reaction solution was centrifuged (12,000×*g*, 15 min, 4 °C) and the supernatant was taken for MDA concentration analysis.

### Immunohistochemistry stain (IHC stain)

After sacrifice, brain tissue was quickly immersed in 10% formalin, embedded in paraffin, and sectioned (3–5 μm/slice). Place the finished tissue sections on Superfrost-coated slides and bake overnight at 45 °C. Sections were deparaffinized in xylene 3 times for 10 min each. Then put it into 95%, 95%, 85%, 75%, 65%, 55% ethanol solution to remove xylene. Wash 3× with TBS solution for 5 min each time to achieve the effect of backwater. Immerse the sections in 10 mM sodium citrate, heat at 100 °C for 30 min to renature the antigen, and then cool. Immediately after completion, immerse the slices in room temperature TBS solution for 10 min. Sectioned tissue was surrounded using a boundary pen (DakoCytomation Pen, Glostrup, Denmark). Then a 3% H_2_O_2_ was added for 10 min. It was washed once more with TBS solution and blocked with 10% horse serum-PBS-T solution for 1 h. Discard blocking solution and add diluted primary antibody for 4 h at 37 °C. The secondary antibodies were added and incubated at 37 °C for 1 h. Wash once with TBS solution and add secondary antibody for 1 h.

### Western blot analysis

Firstly, polyvinylidene fluoride (PVDF) membrane was soaked in methanol. Remove the gel after SDS electrophoresis. Rinse the gel and PVDF membrane with ultrapure water. Sequentially wetted 1-step transfer buffer (84731, Thermo Fisher Scientific, USA) stacks from positive to negative: 7 filter papers (8 × 10.5 cm), PVDF transfer membrane wetted with methanol, electrophoresis gel, 7 filter paper and were transferred with 1.3 A of methanol for 10 min. (Thermo Scientific Pierce G2 Quick Blotter, USA). The membrane was then incubated with blocking solution (3% skim milk) for 1 h, and the proteins were immunodetected using primary antibody, secondary antibody, and chemiluminescent HRP substrate (Millipore). Finally, Chemi Capture software was used for analysis, and Array Analysis software was used for quantitative analysis to compare the expression levels of target proteins in each tissue.

### Statistical analysis

All experiments were performed in triplicate and experimental results were expressed as mean ± standard deviation. One-way ANOVA of SPSS system was used for statistical processing, and Duncan’s multiple range test was used to compare the differences between groups. *P* < 0.05, *p* < 0.01 and *p* < 0.001 indicate significant differences.

## Results

### Morris water maze test to analyze memory behavior

**Fig. 1:**
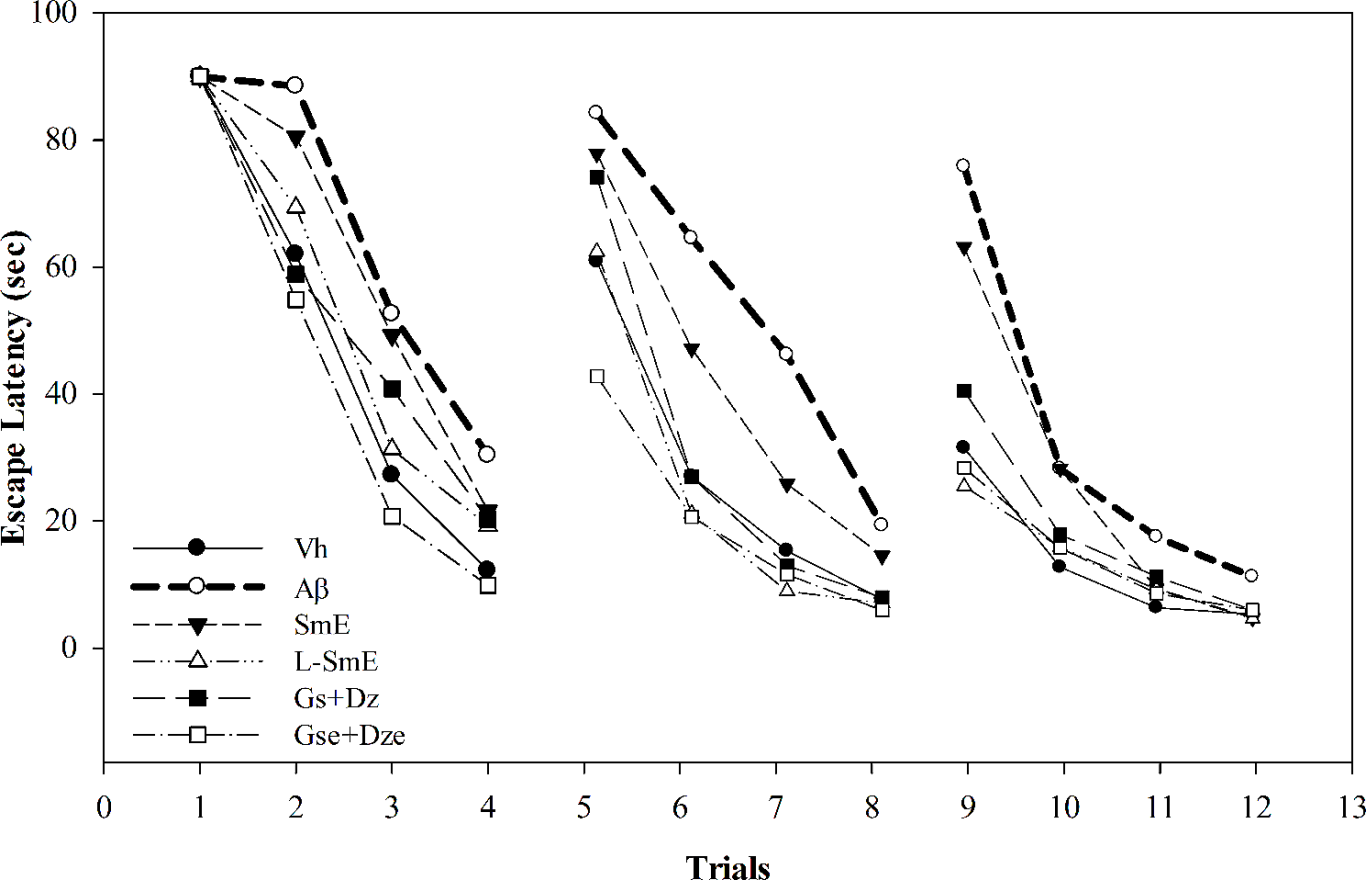
Effect of ethanol extracts of soy milk (SmE) and NTU 101-fermented soy milk (L-SmE) on the memory and learning ability of Aβ40-infused rats in the reference memory task. Groups of rats were infused with vehicle solution (Vh group) or Aβ40 solution (Aβ group) through i.c.v. injection without the administration of test materials. Other Aβ40-infused rats injected with Aβ40 were administered NTU101-fermented soybean milk ethanol extract (L-SmE; 116.4 mg/kg/day, L-SmE group) and soybean milk ethanol extract (SmE; 116.4 mg/kg/day, SmE group), genistin + daidzin (Gs + Dz; 0.729 + 0.789 mg/kg/day, Gs + Dz group), and genistein + daidzein (Gse + Dze; 0.729 + 0.789 mg/kg/day, Gs + Dz group)

**Fig. 2:**
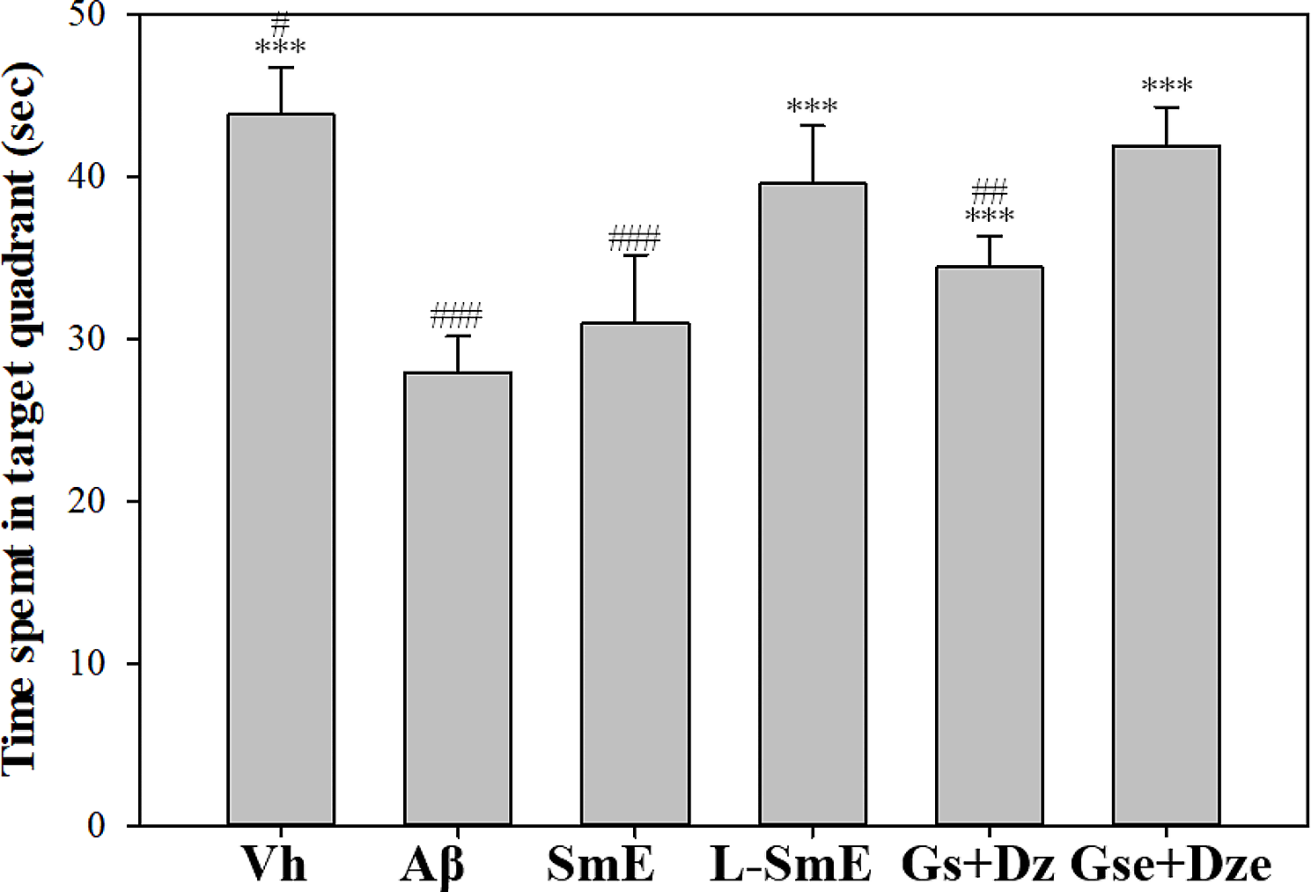
Effect of ethanol extracts of Soy milk (SmE) and ethanol extracts of NTU 101-fermented soy milk (L-SmE) on the memory and learning ability the Aβ40-infused rats in the probe test. Groups of rats were infused with vehicle solution (Vh group) or Aβ40 solution (Aβ group) through i.c.v. injection without the administration of test materials. Other Aβ40-infused rats injected with Aβ40 were administered NTU101-fermented soybean milk ethanol extract (L-SmE; 116.4 mg/kg/day, L-SmE group), soybean milk ethanol extract (SmE; 116.4 mg/kg/day, SmE group), genistin + daidzin (Gs + Dz; 0.729 + 0.789 mg/kg/day, Gs + Dz group), and genistein + daidzein (Gse + Dze; 0.729 + 0.789 mg/kg/day, Gs + Dz group). Data are presented as the mean ± standard deviation (*n* = 8). ***Indicate a significant difference *p* < 0.001) compared with Aβ. ^#^ and ^###^ indicate a significant difference (*p* < 0.05, and *p* < 0.001, respectively) compared with L-SmE

The reference memory test involved 12 experiments performed for 3 consecutive days to determine the long-term memory of experimental animals. The results are presented in Fig. 1. In Experiment 1, each group could not find a platform in the third quadrant for a limited time of 90 s. After testing the learning and memory of 1–4, the search time of the Vh, test substance, and pure substance groups (Vh, SmE, L-SmE, Gs + Dz and Gse + Dze) was lower than that of the Aβ group. Trial 5 included the first swim of the second day. The search time decreased for the SmE, L-SmE, Gs + Dz, Gse + Dze and Vh groups. By contrast, the search time of the Aβ group in Trial F did not considerably differ from that in Trial 1. Trials 7 and 8 were the second and third trials conducted on the second day, respectively. The search time of the test substance and Vh groups was lower than that of the Aβ group, and could be seen from the first swim on the third day of trial 9, the search time of the test substance group was greatly shortened. The search time of the test substance group was considerably shorter in this trial. The search time of the Vh group was shorter than that of the Aβ group, indicating that Aβ40 significantly increased the search time of the Aβ group. The search time of the L-SmE and Gse + Dze groups was shorter than that of the SmE and Gs + Dz groups, indicating that deglycosylated isoflavones were more effective than glycosyl isoflavones in reducing the time required by rats to find the platform. On day 26, after the reference memory test, a spatial probe test was performed. During the test, the platform originally placed in the third quadrant was removed, and the time spent in the quadrant where the platform was placed in the original reference memory test was recorded for the entire swimming path. The probe test demonstrates the spatial detection ability and authenticity of the reference memory test, and its findings can validate the correctness of the results of the reference memory test and help exclude the effects factors with uncertainty, such as luck. The Aβ group stayed in the target quadrant significantly less than did the Vh group (*p* < 0.001; Fig. 2).

**Fig. 3:**
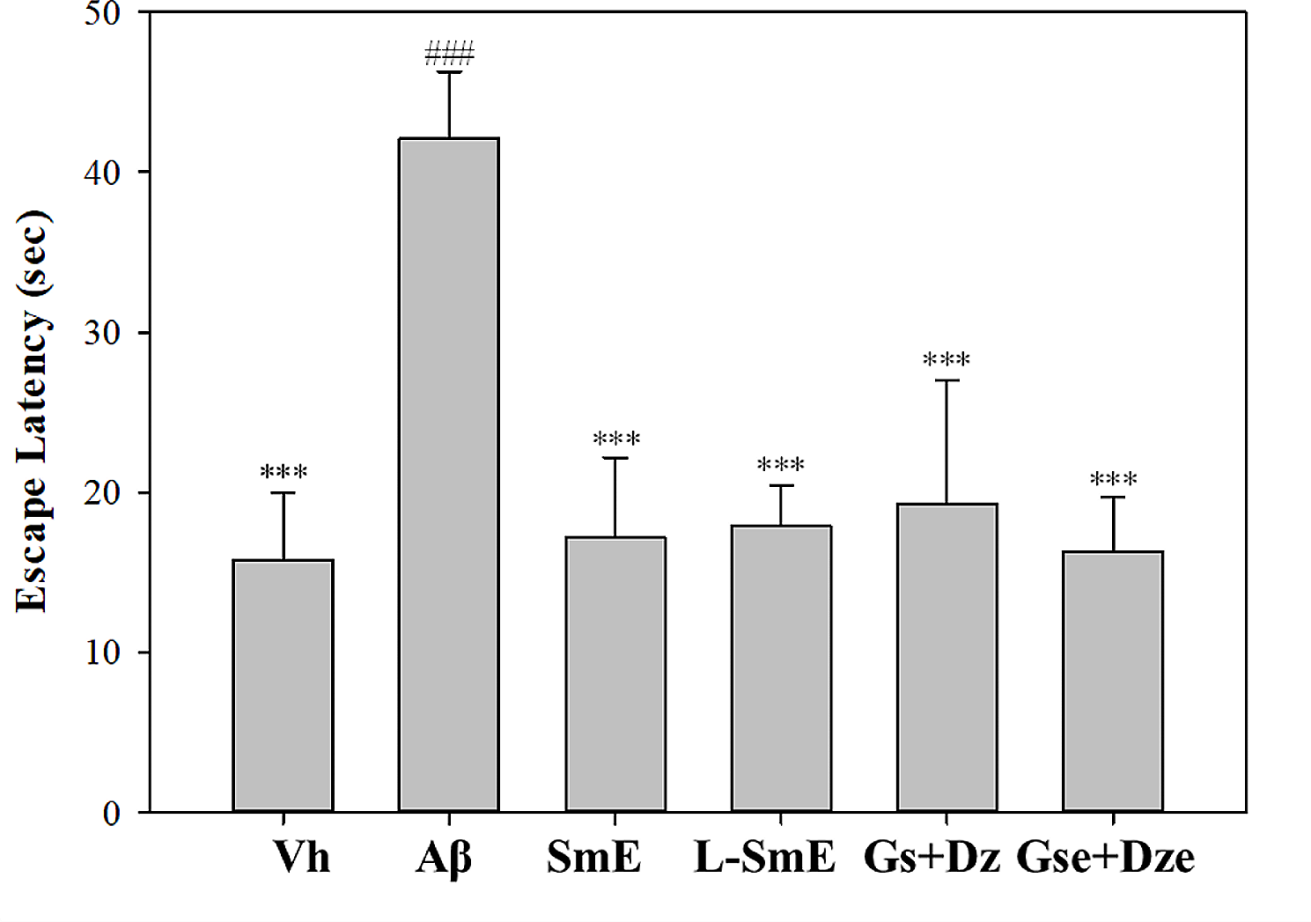
Effect of ethanol extracts of Soy milk (SmE) and ethanol extracts of NTU 101-fermented soy milk (L-SmE) on the memory and learning ability the Aβ40-infused rats in the working memory task. Groups of rats were infused with vehicle solution (Vh group) or Aβ40 solution (Aβ group) through i.c.v. injection without the administration of test materials. Other Aβ40-infused rats injected with Aβ40 were administered NTU101-fermented soybean milk ethanol extract (L-SmE; 116.4 mg/kg/day, L-SmE group), soybean milk ethanol extract (SmE; 116.4 mg/kg/day, SmE group), genistin + daidzin (Gs + Dz; 0.729 + 0.789 mg/kg/day, Gs + Dz group), and genistein + daidzein (Gse + Dze; 0.729 + 0.789 mg/kg/day, Gs + Dz group). Data are presented as the mean ± standard deviation (*n* = 8). ***Indicate a significant difference (*p* < 0.001) compared with Aβ. ^###^Indicate a significant difference (*p* < 0.001) compared with L-SmE

The L-SmE group exhibited more significant improvement in the reference memory ability than did the SmE group (*p* < 0.001), and the Gse + Dze group demonstrated more significant improvement than did the Aβ group (*p* < 0.001). More improvement in the reference memory ability test was noted in the the Gse + Dze group than in the Gs + Dz group. The Gse + Dze group exhibited the most improvement, followed by the L-SmE group. Although the improvement noted in the Gs + Dz group was not as favorable as that observed in the L-SmE and Gse + Dze groups, the Gs + Dz group exhibited significant improvement in spatial detection ability (*p* < 0.001). No significant decrease in spatial detection ability was noted in the SmE group (*p* > 0.05). The L-SmE and Gse + Dze groups demonstrated significantly more improvement than did the SmE and Gs + Dz groups (*p* < 0.05). The Gse + Dze group exhibited the most improvement, followed by the L-SmE group. Although the improvement noted in the Gs + Dz group was not as favorable as that in the L-SmE and Gse + Dze groups, the Gs + Dz group exhibited significant improvement in spatial detection ability (*p* < 0.001). No significant decrease in spatial detection ability was noted in the SmE group (*p* > 0.05). The L-SmE and Gse + Dze groups exhibited significantly more improvement in spatial detection ability than did the SmE and Gs + Dz groups (*p* < 0.05). A significant increase in spatial detection ability was observed in the Aβ and Vh groups (*p* < 0.001), indicating that Aβ40 affected the amount of time the Aβ group remained in the target quadrant. The L-SmE group exhibited more significant improvement than did the SmE group (*p* < 0.01). The Gse + Dze group exhibited more improvement in spatial detection ability than did the Gs + Dz group, and the Gse + Dze group demonstrated the most improvement. The working memory test performed on the 27th day indicated the short-term memory ability of the experimental animals (Fig. 3). The results reveal that the Aβ group required more time to find the resting platform than did the Vh group (*p* < 0.001), indicating that the Aβ group had impaired memory and learning ability and that Aβ40 reduced the ability of laboratory animals to find the platform in the working memory test. The working memory ability of the SmE, L-SmE, Gs + Dz, and Gse + Dze groups exhibited significant improvement (*p* < 0.001); their search time did not significantly differ from that of the Vh group (*p* > 0.05).

### Expression of risk factors for AD

**Fig. 4:**
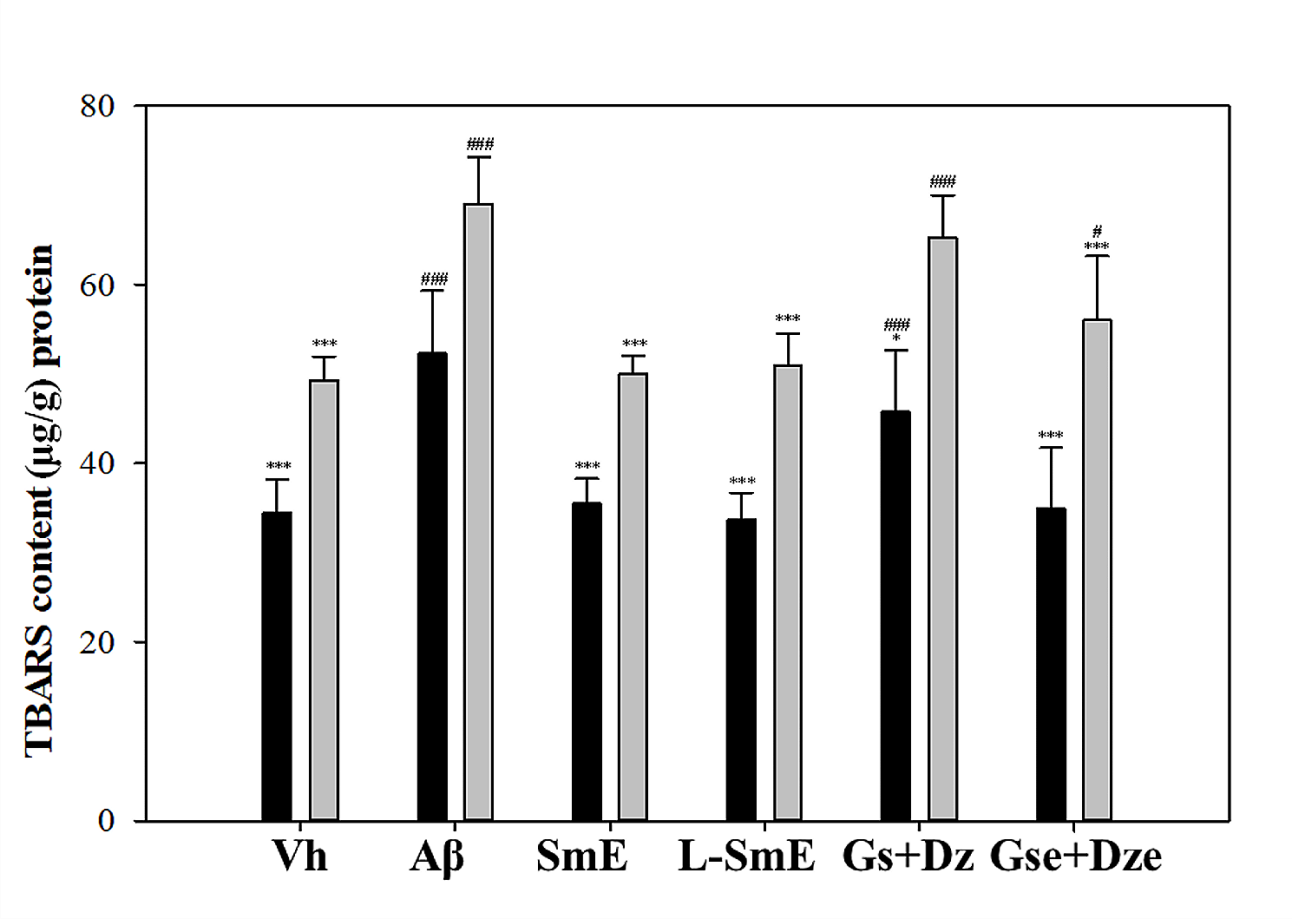
Western blotting and quantitative analysis of the effects of soy milk ethanol extract (SmE) and NTU-101 fermented soy milk ethanol extract (L-SmE) on risk factors in the cortex (**A**) and hippocampus (**B**) of Aβ40-infused rats. Rats injected with Aβ40 were administered NTU101-fermented soybean milk ethanol extract (L-SmE; 116.4 mg/kg/day, L-SmE group), soybean milk ethanol extract (SmE; 116.4 mg/kg/day, SmE group), genistin + daidzin (Gs + Dz; 0.729 + 0.789 mg/kg/day, Gs + Dz group), and genistein + daidzein (Gse + Dze; 0.729 + 0.789 mg/kg/day, Gs + Dz group). Data are expressed as the mean ± standard deviation. *, **, and *** indicate significant differences. The letters indicate significant values compared with Aβ. #, ##, and ### indicate significantly different values compared with L-SmE. All data were analyzed using one-way analysis of variance and Duncan’s multiple test (*p* < 0.05, *p* < 0.01, and *p* < 0.001, respectively)

The abnormal phosphorylation of tau protein results in the formation of double-stranded helical fibers and reduces the ability of microtubules to form an assembly and can lead to the disintegration of an already formed assembly, thus impairing axoplasmic transport and neurotransmitter degradation. This eventually leads to the degeneration and death of neuronal cells (Wu et al. 2015). The p-Tau protein content was significantly higher in the cerebral cortex of the Aβ group than in that of the Vh group (*p* < 0.05; Fig. 4C and D). A significantly increased tau content was noted in the hippocampus of the Aβ and Vh groups (*p* < 0.001). The L-SmE group had a significantly lower p-Tau protein content than did the Aβ group (*p* < 0.01). The L-SmE group had a significant inhibitory effect on the p-Tau protein content of the Aβ group (*p* < 0.01), but had no significant effect on the SmE group (*p* > 0.05). However, the p-Tau protein content tended to be higher in the SmE group than in the Aβ group. In the hippocampus, the L-SmE and SmE groups had a significantly lower p-Tau protein content (*p* < 0.01 and *p* < 0.05, respectively). However, no difference in the p-Tau content was noted between the L-SmE and SmE groups (*p* > 0.05). Both the Gs + Dz and Gse + Dze groups had a significantly lower p-Tau protein content in the cortex and hippocampus (*p* < 0.01).

**Fig. 5:**
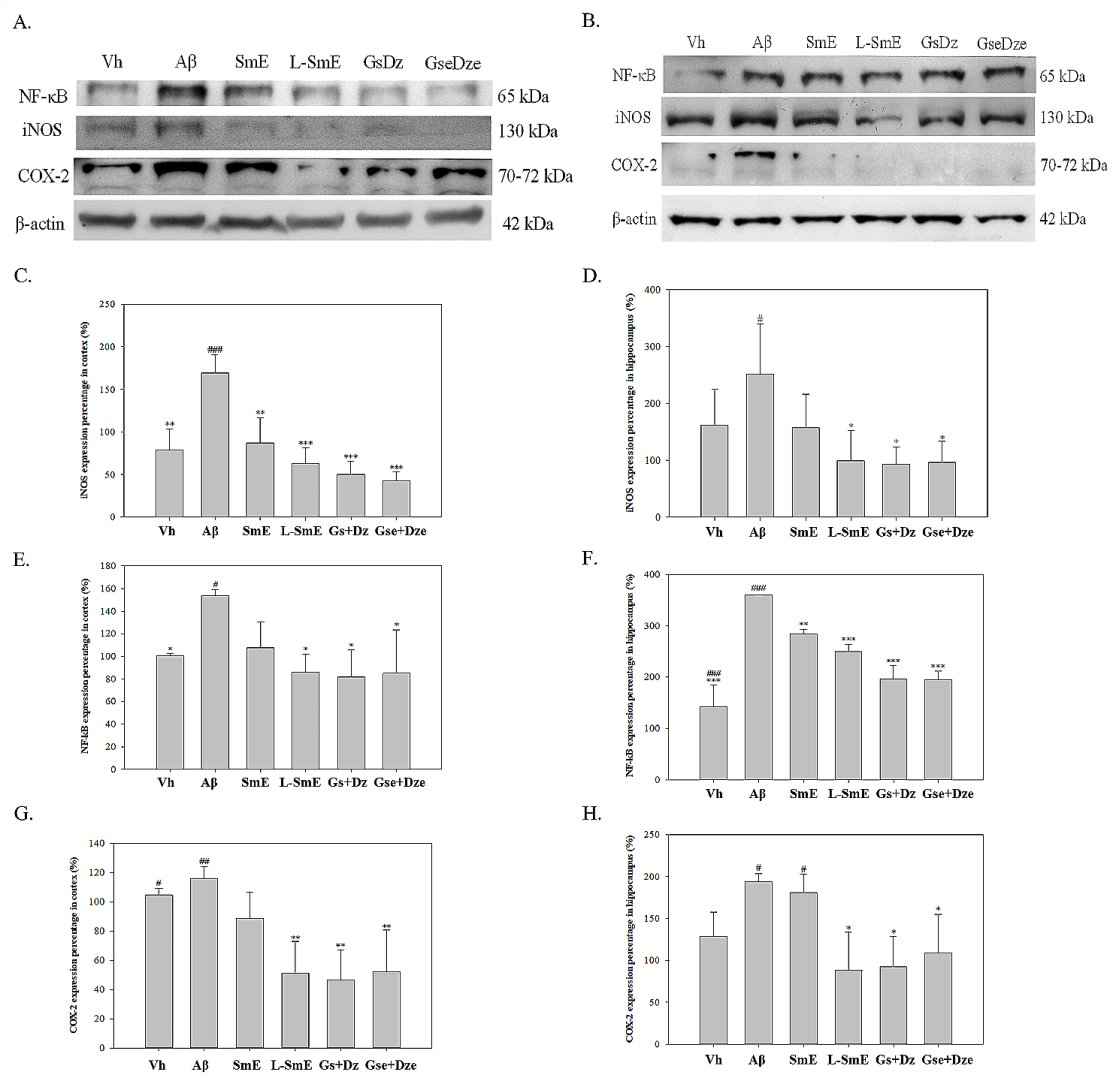
Effect of ethanol extracts of soy milk (SmE) and ethanol extracts of NTU 101-fermented soy milk (L-SmE) on Aβ40 accumulation in the hippocampus of Aβ40-infused rats. Immunohistochemical staining was performed using the nonbiotin hydrogen peroxidase kit. Aβ40 accumulation in the hippocampus was monitored microscopically and revealed brown staining. The nucleus of the section was stained with hematoxylin, as indicated by blue staining. Two groups of the rats were infused with vehicle solution (Vh group) or Aβ40 solution (Aβ group) without the administration of test materials. Rats injected with Aβ40 were administered NTU101-fermented soybean milk ethanol extract (L-SmE; 116.4 mg/kg/day, L-SmE group), soybean milk ethanol extract (SmE; 116.4 mg/kg/day, SmE group), genistin + daidzin (Gs + Dz; 0.729 + 0.789 mg/kg/day, Gs + Dz group), and genistein + daidzein (Gse + Dze; 0.729 + 0.789 mg/kg/day, Gs + Dz group)

Apo E is a low-density lipoprotein transporter and has a high affinity for Aβ (Schmidt et al. 2014). Thus, Apo E leads to the accelerated deposition of Aβ, which, in turn, binds to v-LDL, resulting in a decrease in lipid metabolism in blood and increasing the risk of AD (Rohn et al. 2014). The results are depicted in Fig. 4E and F. In the cerebral cortex and hippocampus, the Aβ and Vh groups exhibited a significantly higher apo E protein content (*p* < 0.05). The L-SmE group had a significantly lower apo E protein content in the hippocampus (*p* < 0.05) but not in the cerebral cortex (*p* > 0.05). The SmE group had a higher apo E protein content in the cerebral cortex than did the Aβ group. However, the apo E protein content in the hippocampus was significantly lower in the SmE group than in the Aβ group (*p* < 0.05). The apo E protein content did not differ between the SmE and L-SmE groups (*p* > 0.05). However, the apo E protein contents in the hippocampus of the SmE and L-SmE groups were significantly lower than that of the Aβ group (*p* < 0.05), but there was no improvement in the cortex (*p* > 0.05). A small amount of Aβ can be detected in human plasma and cerebrospinal fluid, which is a normal metabolic phenomenon. However, the Aβ content in the cerebrospinal fluid of patients with AD is higher, and the clearance capacity of the Aβ content is lower in patients with AD than in healthy individuals (Sun et al. 2015). Aβ can be deposited around the hippocampus, leading to NBTs and senile plaque deposition (Lloret et al. 2015). The immune response of the Aβ group was significantly higher than that of the Vh group, and the protein deposition area on the hippocampus was larger for the Aβ group than for all the groups fed with the test substances (Fig. 5); this result indicates that the Aβ protein content was higher in the Aβ group than in all the other groups. In addition, the SmE group exhibited protein deposition only in the area close to the midbrain; the protein deposition area of the SmE group was significantly smaller than that of the Aβ group. In the L-SmE group, fewer deposition reactions occurred, protein spots were no longer visible near the hippocampus, and only sporadic reactions appeared in the midbrain. More protein deposition reactions occurred in the Gs + Dz group than in the Gse + Dze group. This result indicates that glycosyl isoflavones can reduce the deposition of Aβ40 protein in the hippocampus.

### Expression of oxidative inflammatory factors in the brain

**Fig. 6:**
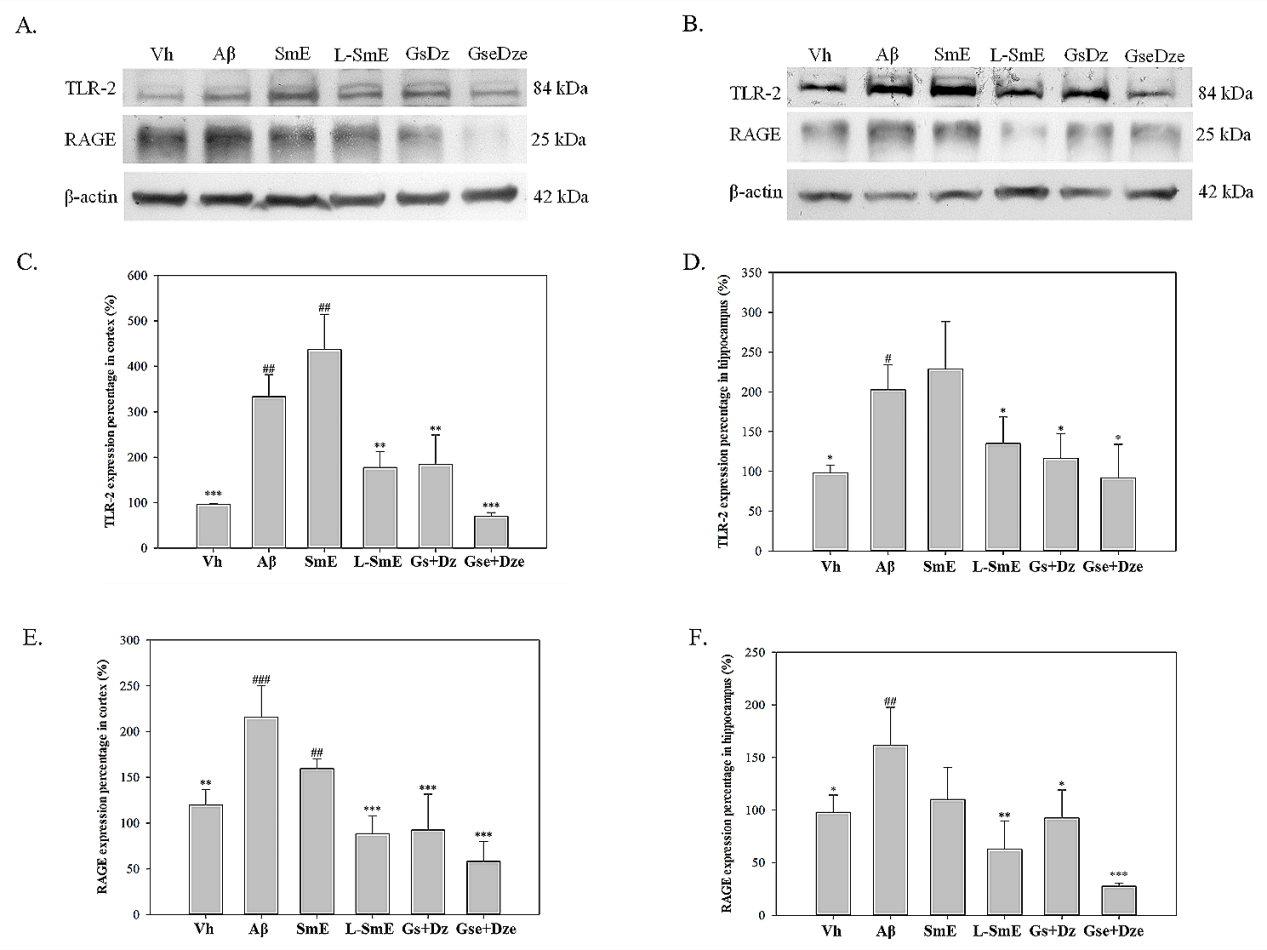
Effect of ethanol extracts of soy milk (SmE) and ethanol extracts of NTU 101-fermented soy milk (L-SmE) on the formation of TBARS in the cortex (black bar) and hippocampus (gray bar) of Aβ40-infused rats. Rats injected with Aβ40 were administered NTU101-fermented soybean milk ethanol extract (L-SmE; 116.4 mg/kg/day, L-SmE group), soybean milk ethanol extract (SmE; 116.4 mg/kg/day, SmE group), genistin + daidzin (Gs + Dz; 0.729 + 0.789 mg/kg/day, Gs + Dz group), and genistein + daidzein (Gse + Dze; 0.729 + 0.789 mg/kg/day, Gs + Dz group). Data are expressed as the mean ± standard deviation. *, and *** indicate significant differences. The letters indicate significant values compared with Aβ. ^#^, and ^###^ indicate significantly different values compared with L-SmE. All data swere analyzed using one-way analysis of variance with Duncan’s multiple test (*p* < 0.05, and *p* < 0.001, respectively)

TBARS is an indicator of oxidative stress. The TBARS content in the Aβ group was significantly higher than that in the Vh group (*p* < 0.001; Fig. 6), indicating that the infusion of Aβ40 in the brain increased the TBARS content. The effect of TBARS was significantly weaker, and the L-SmE and SmE group exhibited a significantly lower TBARS content (*p* < 0.001). The Gse + Dze group had significantly lower TBARS content in the cerebral cortex and hippocampus (*p* < 0.001), and the TBARS content was significantly lower in the Gse + Dze group than in the L-SmE group (*p* < 0.05). The Gs + Dz group exhibited significantly lower TBARS content only in the cerebral cortex. The TBARS content in the Gse + Dze group was lower than that in the Gs + Dz group. TNF-α stimulates 1κB kinase to release p50 and p65 and aggregate into NF-κB. The released NF-κB enters nuclear activation–related genes and then translates inflammatory substances, including the inflammatory response proteins COX-2 and iNOS (Lin et al. 2013).

**Fig. 7:**
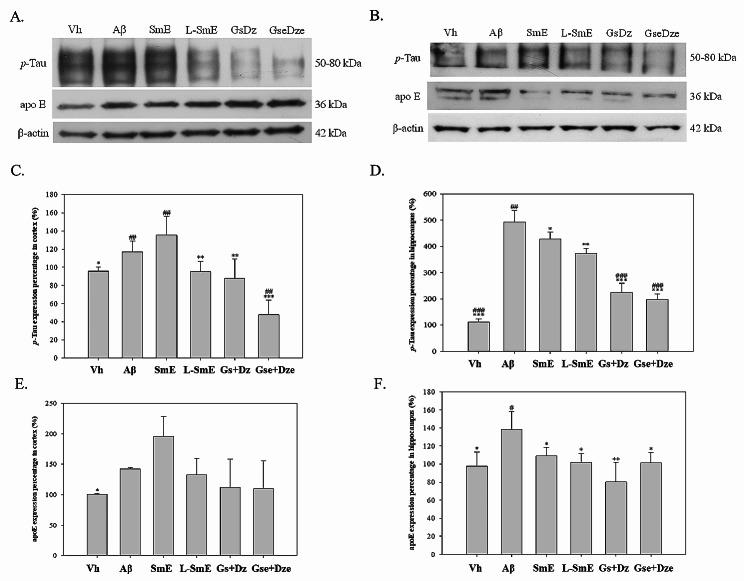
Western blotting and quantitative analysis of the effects of soymilk ethanol extract (SmE) and NTU 101-fermented soymilk ethanol extract (L-SmE) on proinflammatory factors in the cortex (**A**) and hippocampus (**B**) of Aβ40-infused rats. Rats injected with Aβ40 were administered NTU101-fermented soybean milk ethanol extract (L-SmE; 116.4 mg/kg/day, L-SmE group), soybean milk ethanol extract (SmE; 116.4 mg/kg/day, SmE group), genistin + daidzin (Gs + Dz; 0.729 + 0.789 mg/kg/day, Gs + Dz group), and genistein + daidzein (Gse + Dze; 0.729 + 0.789 mg/kg/day, Gs + Dz group). Data are expressed as the mean ± standard deviation *, **, ***. Different letters indicate significant values compared with Aβ. #, ###. Different letters indicate significantly different values compared with L-SmE. All data were analyzed using one-way analysis of variance with Duncan’s multiple test (*p* < 0.05, *p* < 0.01, and *p* < 0.001, respectively)

The Aβ group had a significantly higher NF-κB protein content than did the Vh group in the cerebral cortex and hippocampus (*p* < 0.05; Fig. 7C and D), indicating that the infusion of Aβ40 in the brain may lead to an increase in NF-κB protein content. The NF-κB protein content was significantly lower (*p* < 0.05) in the Aβ group than in the L-SmE group but not the SmE group (*p* > 0.05). In the hippocampus, both the L-SmE and SmE exhibited a significantly lower NF-κB protein content than did the Aβ group (*p* < 0.001 and *p* < 0.01, respectively). Although the SmE group had a lower NF-κB protein content than did the L-SmE group, the difference was nonsignificant (*p* > 0.05). Although the Gs + Dz and Gse + Dze groups both had a significantly lower NF-κB protein content (*p* < 0.05), it did not differ from that of the L-SmE group (*p* > 0.05). A large amount of iNOS produces a large amount of NO, thus leading to the availability of a large number of free radicals that attack cells and tissues and aggravate their inflammatory response. The Aβ and Vh groups exhibited a significantly higher iNOS protein content in the cerebral cortex (*p* < 0.01) but not in the hippocampus (*p* > 0.05; Fig. 7E and F). The iNOS protein content tended to be higher in the Aβ group. The L-SmE group had a significantly lower iNOS protein content in the cortex (*p* < 0.001), but no significant difference in the iNOS protein content was noted between the L-SmE and other groups (*p* > 0.05). In the hippocampus, the L-SmE group exhibited a significantly lower iNOS protein content than did the Aβ group (*p* < 0.05). The Gs + Dz and Gse + Dze groups had a significantly lower iNOS protein content in the cortex than did the Aβ group (*p* < 0.001). However, the L-SmE group did not exhibit a significantly lower iNOS protein content (*p* > 0.05). The Gs + Dz and Gse + Dze groups had a significantly lower iNOS protein content in the hippocampus than did the Aβ group (*p* < 0.05); however, the iNOS protein content did not significantly differ between the Gs + Dz and Gse + Dze groups and the L-SmE group (*p* > 0.05). COX-2 increases blood flow by stimulating vasodilators and histamine to dilate blood vessels and activates macrophages or other immune cells to flood the inflamed tissue to exacerbate the inflammatory response (Medeiros et al. 2010).

The COX-2 protein content in the L-SmE group was significantly higher than that in the Aβ group (*p* < 0.01; Fig. 7G and H). The COX-2 protein content was significantly higher in the Gs + Dz and Gse + Dze groups (*p* < 0.01 and *p* < 0.05, respectively). The Vh group had a lower COX-2 protein content in the hippocampus than did the Aβ group; however, this difference was nonsignificant (*p* > 0.05). The L-SmE group exhibited a significantly lower COX-2 protein content than did the Aβ group (*p* < 0.05), whereas the SmE group did not exhibit a significantly lower COX-2 protein content (*p* > 0.05). The pure substance group had a significantly lower COX-2 protein content (*p* < 0.05); however, the L-SmE group did not have a significantly lower COX-2 protein content (*p* > 0.05).

### Expression of astrocyte and microglia activators

Astrocyte and microglia activators are receptors for advanced glycation end products (RAGE) and Toll-like receptor 2 (TLR-2). RAGE is a transmembrane receptor immunoglobulin that binds to advanced glycation end products (AGEs) or Aβ (Walker et al. 2015). TLR-2 is often present on the cell surface of astrocytes and microglia, and its main function is to recognize antigens and trigger innate immune responses. After binding to the antigen, TLR-2 releases inflammatory substances (Ebert et al. 2015).

**Fig. 8:**
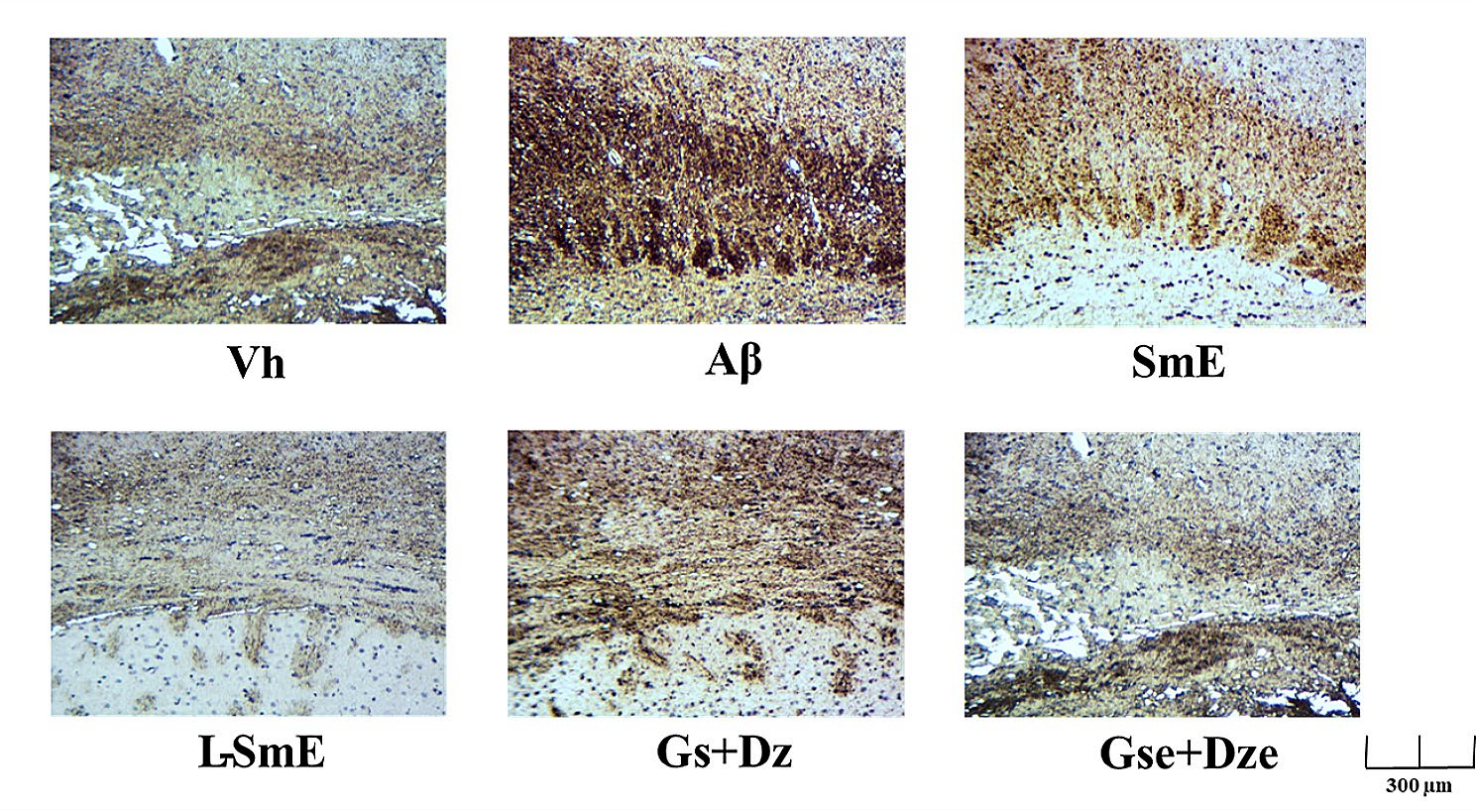
Western blotting and quantitative analysis of the effects of soy milk ethanol extract (SmE) and NTU 101-fermented soy milk ethanol extract (L-SmE) on Aβ40-infused rat cortex (**A**) and hippocampus (**B**) astrocytes and microglia. Rats injected with Aβ40 were administered NTU101-fermented soybean milk ethanol extract (L-SmE; 116.4 mg/kg/day, L-SmE group), soybean milk ethanol extract (SmE; 116.4 mg/kg/day, SmE group), genistin + daidzin (Gs + Dz; 0.729 + 0.789 mg/kg/day, Gs + Dz group), and genistein + daidzein (Gse + Dze; 0.729 + 0.789 mg/kg/day, Gs + Dz group). Data are expressed as the mean ± standard deviation *, **, ***. Different letters indicate significant values compared with Aβ. #, ##, ###. Different letters indicate significantly different values compared with L-SmE. All data were analyzed using one-way analysis of variance with Duncan’s multiple test (*p* < 0.05, *p* < 0.01, and *p* < 0.001, respectively)

The Aβ group had higher LR-2 and RAGE protein contents than did the Vh group (Fig. 8). The SmE group had higher TLR-2 and RAGE protein content. Even in the hippocampus, the TLR-2 protein content was higher in the SmE group than in the Aβ group, whereas the L-SmE group exhibited a significantly lower TLR-2 protein content. The TLR-2 protein content was lower in the Gse + Dze group than in the Gs + Dz group.

## Discussion

This study investigated the effect of NTU101 fermented and unfermented soy milk on AD. We fed rats with different types of pure substances, namely genistein, daidzein, and equol, to explore the main functional components of fermented soybean milk. We also compared the effectiveness between glycosylated and postfermentation deglycosylated isoflavones in improving AD. Aβ is a major risk factor for memory and learning deficits in patients with AD. The Aβ content is higher in the brain of patients with AD, and metabolic efficiency is lower in both patients with AD and older individuals. They exhibit cranial nerve accumulation and neural network disruption, which are related to cognitive dysfunction (Elman et al. 2016). An animal model of AD can be successfully established by injecting Aβ40 into the lateral ventricle of the rat brain (Qian et al. 2022). Aβ deposits injected into the brain cause oxidative stress and inflammatory responses that damage neurons and ultimately impair memory and learning (Amini et al. 2015). Neuropathologically, Aβ and NBTs in the hippocampus form senile plaques that are deposited in the cerebral cortex (Roberts et al. 2012).

Genistein, an aglycone isoflavone, is called phytoestrogen because its function and conformation are similar to those of estrogen. Genistein can ameliorate neurodegenerative diseases and thus is used in hormone replacement therapy. Genistein does not cause the unrestricted proliferation of cells, leading to increased cancer risk (Setchell 2006). Genistein exerts neuroprotective effects. Genistein can effectively reduce the expression of oncogenes, such as P53 and CHD5, and inhibit the proliferation of blood vessels, thus inhibiting the generation of neuroblastoma (Sharifi-Rad et al. 2021). Daidzein also exerts a neuroprotective effect; it can promote axon growth, stimulate apo E production in mice congenitally lacking the lipid carrier protein, and improve stroke caused by lipid accumulation. Long-term consumption of daidzein exerts a protective effect on chronic diseases of the nervous system (Kim et al. 2015). In addition, inhibition of the splicing pathway of Aβ prevents the β-amyloid precursor protein from splicing into Aβ through the amyloidogenic pathway, thus preventing it from generating and accumulating amyloid plaques (Ahmad et al. 2015), activating nerve conduction pathways, increasing the acetylcholine level, reducing the acetylcholinesterase level to prevent N-methyl-D-aspartate receptors from opening ion channels, and causing excessive calcium damage in neurons (Bagheri et al. 2012). In addition, daidzein also can prevent the release of oxidative inflammatory substances by astrocytes and microglia; increase the production of antioxidative enzymes, such as SOD and catalase; and inhibit the production of TNF-α and IL-1β. The expression of proinflammatory factors can prevent or inhibit AD (Bagheri et al. 2011).

The Morris water maze test is commonly used to evaluate the memory and learning ability of rodents. This test does not require animal training, making it impossible for animals to use body odor recording paths (Iivonen et al. 2003). The reference memory test is used to evaluate long-term memory. The spatial detection test is used to validate the findings of the reference memory test, and the working memory test is used to evaluate short-term memory. Assessment of spatial cognitive impairment or memory impairment caused by AD is an appropriate method (Laczó et al. 2010). The results of the water maze test reveal that the Aβ group spent more time from the first to the third day searching the platform than did the Vh and test substance groups. In the space exploration test, the swimming path of the Aβ group presented an irregular surrounding pool shape, which was significantly different from that of the group fed with the test substances (*p* < 0.001). In the working memory test, which was performed to evaluate short-term memory, although the Aβ group found the platform in the pool, its search time was significantly longer than that of the Vh group. These results indicate that the injection of Aβ40 in the posterior ventricle on the lateral side of the rat brain caused impaired memory and learning. The L-SmE group required considerably less time to findi the platform in the reference memory test on the second day than did the soymilk alcohol extraction group, with a difference of more than 30 s on the third day. In the spatial detection test, the residence time of the L-SmE group in the target quadrant was almost identical to that of the Vh and Aβ groups. Fermented soybean milk improved the memory and learning ability more effectively than did unfermented soybean milk. The Gse + Dze group exhibited the same trend as did the Gs + Dz group, and this result can be attributed to the use of fermented soybean milk. Unglycosylated isoflavones are converted into deglycosylated isoflavones, and deglycosylated isoflavones exhibit higher physiological activity. In the space detection test, the fermented soybean milk or deglycosylated isoflavone group exhibited more improvement.

These findings are similar to those of other studies. Ahmed used tempeh, a fermented soybean food, as a test substance to inhibit BACE activity. The ability of tempeh to inhibit BACE activity is superior to that of nondeglycosylated isoflavones, and tempeh is also more effective than nondeglycosylated isoflavones in scavenging DPPH free radicals (Ahmad et al. 2015). Aβ induces oxidative stress, causing increased production of superoxide and lipid peroxides in neurons. Astrocytes and microglia release peroxides when they bind to Aβ fibrils, including NO, superoxide anion, and other free radicals that attack nerve cells and cause their death (Magalingam et al. 2018).

Numerous studies have reported the antioxidative activity of soy isoflavones. For example, Zhang et al. (2007) demonstrated that soy isoflavones can reduce oxidative stress and inhibit TNF-α protein expression, thus reducing the influx of calcium ions into cells and leading to their death.

The TBARS content was significantly higher in the Aβ group than in the Vh group (*p* < 0.001) because of the intracerebral infusion of Aβ40. At the same protein concentration, the TBARS content was higher in the hippocampus than in the cerebral cortex. A possible reason is that abnormal Aβ40 metabolism and deposition in the hippocampus produce risk factors, such as NBT formation and senile plaque deposition (Rather et al. 2021). The alcoholic extracts of both fermented and unfermented soybean milk significantly reduced the TBARS content (*p* < 0.001); however, no significant difference was noted in their ability to reduce the TBARS content (*p* > 0.05). Isoflavones exhibit strong antioxidative activity (Bagheri et al. 2013). Fermentation and deglycosylation processes limited their antioxidative effect; thus, no significant difference was noted between the L-SmE and SmE groups or between the SmE and pure substance groups. Although the deglycosylated isoflavone group exhibited a stronger inhibitory effect on the TBARS content, it was still not significant (*p* > 0.05).

Inflammation is associated with several neurodegenerative diseases and might lead to the increased production and accumulation of Aβ. After being activated by RAGE, astrocytes and microglia release peroxides and inflammatory substances to attack nerve cells and cause their death (Rather et al. 2021). Mitochondria may become dysfunctional, which can eventually lead to cell death due to excessive free radicals attacking bilayer phospholipids (Sinha et al. 2013). Activation of proinflammatory factors, such as TNF-α, NF-κB, and IL-1β, causes changes in inflammatory pathways. After NF-κB enters the nucleus, it is converted to COX-2 and iNOS (Lin et al. 2013). COX-2 can dilate blood vessels to increase blood flow and activate macrophages to flood inflamed tissues (Medeiros et al. 2010).

iNOS induces the production of a large amount of NO, which can easily combine with oxygen to generate a large number of free radicals that attack cell tissues and cause damage (Song et al. 2014). The results of this study reveal that the levels of the proinflammatory factors NF-κB, iNOS, and COX-2 in the cerebral cortex and hippocampus were significantly higher in the Aβ group than in the Vh group (*p* < 0.05), indicating that the infusion of Aβ40 in the brain may lead to the production of a large number of proinflammatory factors and cause a severe oxidative inflammatory response. The ethanolic extract of fermented soymilk significantly improved the levels of the three proinflammatory factors (*p* < 0.05), whereas the ethanolic extract of unfermented soymilk inhibited NF-κB and iNOS protein expression (*p* < 0.05) but had no effect on COX-2 expression (*p* > 0.05).

NTU101 fermented soybean milk resulted in more improvement in oxidative stress than did unfermented soybean milk. Compared with the Aβ group, the groups receiving glycosylated isoflavones and deglycosylated isoflavones demonstrated significant improvement (*p* < 0.05). However, no difference between the glycosylated isoflavone and deglycosylated isoflavone groups was noted. Genistein, genistein, daidzein, and daidzein have similar structures and can be interconverted in vivo (Toktay et al. 2020); thus, they can exert similar effects. However, the absorption time of NTU101 fermented and unfermented soybean milk may be different from that of glycosyl isoflavones because deglycosylated isoflavones are more easily absorbed. Therefore, the effect of fermented soybean milk is stronger than that of unfermented soybean milk.

In terms of AD-related factors, Aβ caused a significant increase in the tau protein and apo E content (*p* < 0.05), indicating that the intracerebral infusion of Aβ40 can effectively induce AD symptoms. The ethanolic extract of fermented soymilk significantly improved Aβ infusion (*p* < 0.05), indicating that NTU101 fermented soymilk can alleviate AD by reducing AD-related factors. Genistein, a functional component of fermented soybean milk, can improve AD. Genistein reduced the β-secretase mRNA level and prevented the splicing of APP into Aβ, thus preventing AD in ovariectomized and estrogen-deficient mice (Li et al. 2013). In addition, genistein can effectively reduce β-secretase activity and the DPPH free radical scavenging ability (Ahmad et al. 2015). These results indicate that fermented soy milk can reduce the deposition of Aβ40 in the brain, thus reducing the expression of risk factors and alleviating AD; the active ingredient is derived from deglycosylated isoflavones.

This study performed tissue immunostaining to evaluate the deposition of Aβ40 in the cortex and hippocampus of experimental animals in each group. The Aβ group had the highest protein content on the slice map, which is consistent with the results of studies indicating that Aβ can be deposited around the hippocampus, leading to NBT and senile plaque deposition (Lloret et al. 2015). In the group fed with the ethanol extract of unfermented soybean milk, protein deposition was noted only near the midbrain, which was significantly lower than that in the Aβ group. After feeding with the ethanol extract of fermented soy milk, we did not observe protein spots near the hippocampus, but sporadic protein deposition appeared in the midbrain, indicating that the ethanol extract of fermented soy milk was more effective than the ethanol extract of unfermented soy milk in removing Aβ40 deposited around the hippocampus. The same results were observed in the group fed with the pure substance. The Gs + Dz group has more protein deposits than did the Gse + Dze group. Fermented soybean milk and deglycosylated isoflavones reduced the Aβ40 protein content deposited in the hippocampus, demonstrating that deglycosylated isoflavones can reduce the Aβ40 protein content deposited in the hippocampus more effectively than can glycated isoflavones.

**Fig. 9:**
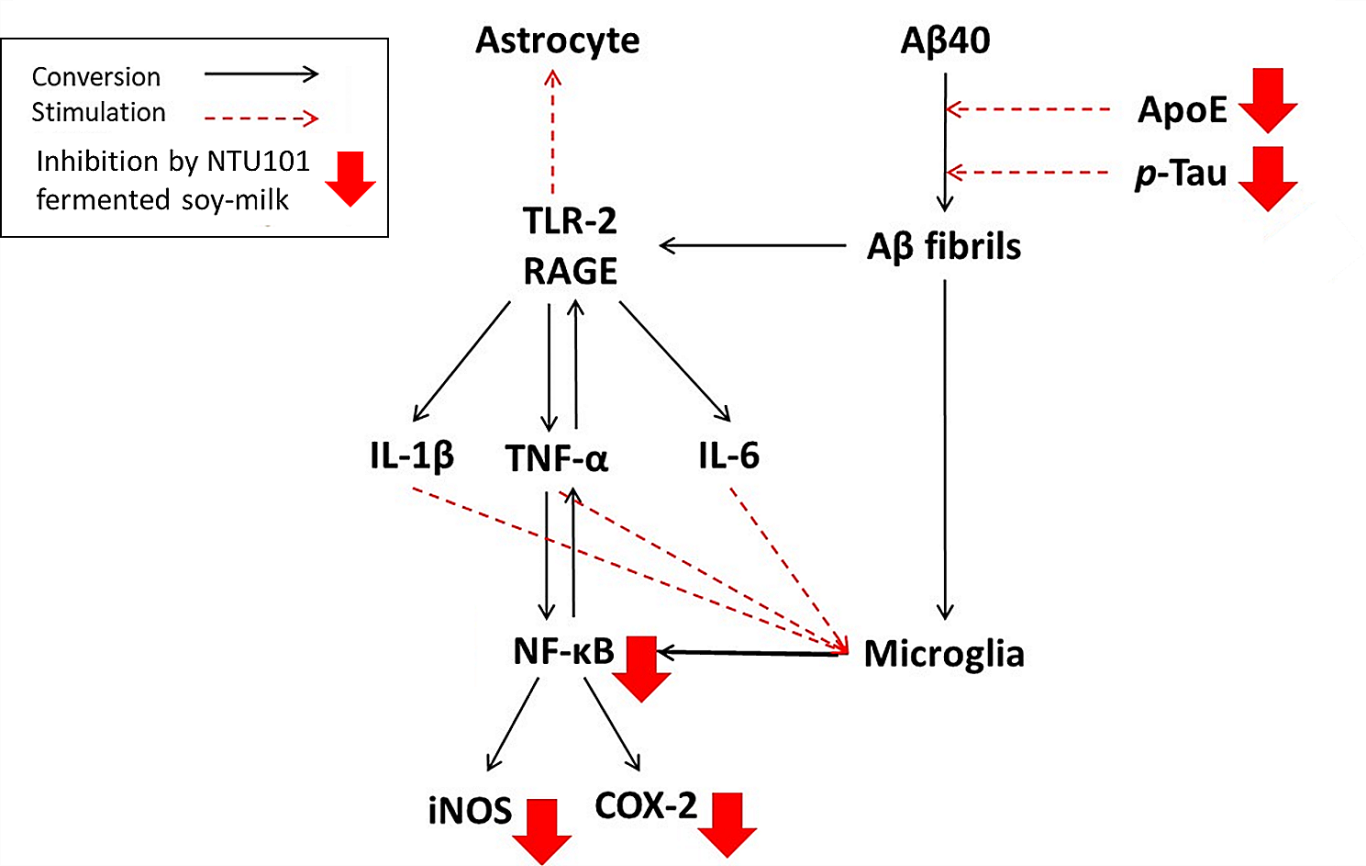
Mechanisms of NTU101-fermented soy-milk in the regulation of the formation of Aβ and proinflammatory response in Aβ40-induced Alzheimer’s disease

In astrocytes, the polysynaptic configuration is formed after contact with the blood vessel wall, which can tightly connect the blood vessel wall in the brain and become a crucial support cell of the central nervous system. Both microglia and astrocytes provide nutrients required by nerve cells for survival and contribute to the recovery of neurotransmitter substances. However, when the brain is in a state of inflammation, inflammatory substances are released because of the activation of RAGE and TLR-2 (Otazu et al. 2021). The Aβ group had higher TLR-2 and RAGE protein content than did the Vh group, indicating that the infusion of Aβ40 in the brain may cause subsequent inflammatory responses due to the activation of RAGE and TLR-2. Western blotting revealed that the protein content of TLR-2 and RAGE was higher in the SmE group, and the protein content of TLR-2 in the hippocampus was even higher than that in the Aβ group. On the contrary, the L-SmE group showed a clear downward trend. In the pure substance group, the TLR-2 protein content was lower in the Gse + Dze group than in the Gs + Dz group. Fermented soybean milk can improve the inflammatory response by weakening the effects of astrocyte and microglia-activating and proinflammatory factors. In summary, *L. paracasei* subsp. *paracasei* NTU101 fermented soybean milk was investigated for its effects on AD. Using AD model rats infused with Aβ40 for 28 days, we found that NTU101 fermented soymilk notably improved AD symptoms. Key findings include the reduction of oxidative inflammation, decreased risk factor expression for tau and apoE proteins, less Aβ40 deposition around the hippocampus, and lowered TLR-2 and RAGE protein expression in astrocytes and microglia (Fig. 9). Additionally, there was a significant enhancement in memory and learning abilities. This study highlights the potential of NTU101 fermented soybean milk as a therapeutic agent for AD, demonstrating its multifaceted benefits.

## Data Availability

All data included in this study are available upon request by contacting the corresponding author.
